# The Aging GABAergic System and Its Nutritional Support

**DOI:** 10.1155/2021/6655064

**Published:** 2021-04-25

**Authors:** Demetra J. Mills

**Affiliations:** Patent Trial and Appeal Board Biotechnology, 5232 Capon Hill Pl, Burke, VA 22015, USA

## Abstract

Aging is associated with a decline in hormones and an associated decline in GABAergic function and calcium and ion current dysregulation. Neurosteroid hormones act as direct calcium channel blockers, or they can act indirectly on calcium channels through their interaction with GABA receptors. The calcium channel dysfunction associated with hormone loss further leads to an excitatory cell state, which can ultimately lead to cell death. The calcium theory of aging posits that cellular mechanisms, which maintain the homeostasis of cytosol Ca^2+^ concentration, play a key role in brain aging and that sustained changes in Ca^2+^ homeostasis provide the final common pathway for age-associated brain changes. There is a link between hormone loss and calcium dysregulation. Loss of calcium regulation associated with aging can lead to an excitatory cell state, primarily in the mitochondria and nerve cells, which can ultimately lead to cell death if not kept in check. A decline in GABAergic function can also be specifically tied to declines in progesterone, allopregnanolone, and DHEA levels associated with aging. This decline in GABAergic function associated with hormone loss ultimately affects GABAergic inhibition or excitement and calcium regulation throughout the body. In addition, declines in GABAergic function can also be tied to vitamin status and to toxic chemicals in the food supply. The decline in GABAergic function associated with aging has an effect on just about every body organ system. Nutritional support of the GABAergic system with supportive foods, vitamins, and GABA or similar GABA receptor ligands may address some of the GABAergic dysfunction associated with aging.

## 1. Introduction

This article primarily focuses on changes in GABAergic function associated with hormone loss and aging. Changes in GABAergic function and hormone loss are ultimately tied to one another and to changes in calcium regulation in the body [1, 2]. While it is recognized that the human body is a complex symphony of many chemical reactions, one or more than one chemical reaction influencing another, the singling out of *γ*-aminobutyric acid (GABA) and GABAergic functions here is not meant to oversimplify these complex interactions, but to acknowledge the universal importance of GABA in regulating ion channel gating throughout the human body.

As one ages, a patient may notice the appearance of symptoms such as higher blood pressure, anxiety or depressive symptoms, and weight gain to name a few. These symptoms may be associated with hormone loss and its correlated GABAergic dysfunction. Neurosteroids, including hormones, and calcium appear to be the integrated elements of signaling systems in neuronal cells under physiological and pathological conditions [3]. Neurosteroid hormones can act directly as calcium channel blockers. Alternatively, neurosteroids can act as GABA A receptor agonists and show similar properties to GABA amino acid neurotransmitter including anticonvulsant, sedative-hypnotic, analgesic, and anxiolytic effects [3]. Experimental data indicate that the neurosteroid, allopregnanolone, is able to induce changes in cellular calcium homeostasis. The action of hormones on GABA receptors may be excitatory or inhibitory, but it has primarily been found that the action of hormones on GABA receptors is inhibitory. The excitatory action of allopregnanolone on GABA A receptor was prevented not only by inhibition of GABA A receptor, but also by nifedipine, a selective Ca^2+^ channel blocker, suggesting that allopregnanolone can generate membrane depolarization and calcium influx after activation of GABA A receptor [3]. Most neurosteroids with a 3*α*-hydroxyl group within A-ring (mainly 3*α*-reduced metabolites of progesterone and deoxycorticosterone, allopregnanolone, 3*α*, 5*α*-THDOC, or androstenediol) are positive allosteric modulators of GABA AR, enhancing GABA-evoked chloride current. It has been established that, in some cells, increased chloride current led to membrane depolarization and to activation of L-type of voltage-gated calcium channels (VGCC) [3]. GABA may also exhibit nontypical, excitatory effect instead of most popular inhibitory action depending upon its location of action. Hormones, binding to GABA B receptors, may ultimately function as calcium channel blockers through a cascade involving G protein couples. Positive GABA A receptor modulators, including neurosteroids, can inhibit L-type voltage-gated calcium channels (L-VGCCs), which contributes to reduced neuronal excitability [4]. Researchers found that positive allosteric GABA AR modulators reversibly inhibited L-VGCCs in a state-dependent manner. The order of potency of positive allosteric GABA AR modulators found was allopregnanolone > Benzodiazepines > pentobarbital > ethanol [4]. Studies suggest that “allopregnanolone also inhibits presynaptic evoked glutamate release via the inhibition of L-type calcium channels in the medial prefrontal cortex” [4]. Inhibition of presynaptic glutamate release also leads to reduced overall neuron excitability [5].

Dehydroepiandrosterone (DHEA) has been found to be a T-type or transient calcium channel blocker which blocks calcium channels within cardiac and smooth muscle [6]. Progesterone has been shown to be an L-type calcium channel blocker [7]. Vitamin D is also a calcium channel blocker and so is testosterone [8, 9]. Estrogen may be unique in that it can both block and excite varying calcium channels [10, 11]. Estrogen may increase the number of GABA binding sites in the rat nervous system by direct interaction with the GABA receptor gene or genes involved in the metabolism of GABA receptor [12]. Recent studies have demonstrated that estrogen rapidly induces Ca^2+^ influx in hippocampal neurons, which is required for neuroprotection and potentiation of long term potentiation LTP [11]. At the same time, estrogen may limit cell death in the nervous system tissue by inhibiting increases in intracellular free Ca^2+^. Estrogen also prevented glutamate-mediated changes in resting membrane potential and membrane capacitance [13].

Therefore, due to hormone loss and its associated effect on the GABAergic system and calcium dysregulation, a patient may be more likely to need a calcium channel blocker, such as verapamil and amlodipine, to control blood pressure, because their hormones, which naturally function in this manner, are reduced with aging. As explained further herein, hormones such as progesterone and DHEA both directly and indirectly affect the GABAergic system. The GABA receptors have ligand binding sites for steroids, such as hormones [14]. The loss of hormones with aging, through their decline in function as GABA receptor agonists, leads to dysregulation of calcium channels and can lead to cell excitotoxicity and cell death. The failure of the inhibitory function of GABA B receptors in response to neurosteroid hormones or GABA amino acid in particular, to keep calcium channels/calcium regulatory systems in check, further exacerbates the cell excitotoxicity, cell dysfunction, and cell death associated with aging. Also reviewed are the many body systems which may be affected by changes to the GABAergic system in aging and nutritional support for the aging GABAergic system.

## 2. Discussion

### 2.1. GABAergic System

The GABAergic system involves “the biosynthesis and metabolic degradation of gamma amino butyric acid (GABA), its release and interaction with receptors, and its inactivation by high-affinity transport systems in GABAergic and glutamatergic neurons and astrocytes” [15]. The GABAergic system is significantly regulated by hormones, including DHEA, progesterone, and allopregnanolone. GABA is an inhibitory neurotransmitter and ion or voltage-gated calcium channel regulator functioning at GABA receptors.

It is now known that GABAergic system homeostasis is critical to many body systems [16]. GABA is critical to body systems and functions including blood pressure, baroreceptor function, human growth hormone release, weight regulation and feeding, respiratory function, brain function, kidney function, vision, and pancreatic function, among others.

The GABA receptors are a class of receptors that respond to the neurotransmitter GABA, the chief inhibitory compound in the human central nervous system. There are three classes of *γ*-aminobutyric receptors (GABA R), *γ*-aminobutyric A receptor (GABA A), *γ*-aminobutyric B receptor (GABA B), and *γ*-aminobutyric C receptor (GABA C). GABA A receptors are ligand-gated ion channels. GABA B receptors are G protein-coupled receptors, also called metabotropic receptors [17]. GABA BRs also inhibit many voltage-sensitive calcium (Ca) channel (VSCC) subtypes [18].

GABA receptors are broadly expressed in the central nervous system (CNS) representing about 20% of the synapses in cortex, hippocampus, thalamus, and cerebellum, where they “possibly control cognitive functions such as memory, language, and attention” [20]. More particularly, GABA B receptor activation at presynaptic locations suppresses neurotransmitter release by inhibition of voltage-sensitive Ca^2+^ channels [20]. GABA C receptors have been associated with visual processing, regulation of sleep-waking rhythms, pain perception, memory, learning, regulation of hormones, and neuroendocrine gastrointestinal secretion. These receptors are also localized in the retina, thalamus, hippocampus, pituitary, and gastrointestinal tract [20].

**Figure 1:**
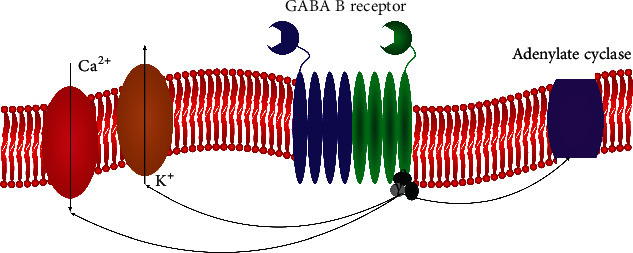
An image of a metabotropic GABA B receptor with its G protein couple (black/gray); adenylate cyclase (purple); potassium channel (beige); and calcium channel (red). This original figure depicts the mechanism by which GABA B receptors influence calcium and potassium channels. Source: [19].

GABA B receptor is a G protein coupled receptor that associates with a subset of G-proteins that in turn regulate specific ion channels, for example, calcium channels, and trigger cAMP cascades. For an illustration of the mechanism of GABA B's influence on calcium channels (see Figure 1). GABA B receptors mediate their inhibitory action by activating inwardly rectifying K+ channels, inactivating voltage-gated Ca^2+^ channels, and inhibiting adenylate cyclase [21]. Postsynaptic receptors induce a slow inhibitory postsynaptic current by gating a special type of “K+-channel, which hyperpolarizes the membrane and shunts excitatory current” [21].

Therefore, when the neurotransmitter GABA attaches to the GABA receptor, it acts as the chief inhibitory neurotransmitter in the central nervous system [20]. GABA's principal role is reducing neuronal excitability throughout the nervous system [21]. GABA B receptor activation by GABA and other ligands, including hormones, induces a conformational change in the receptor. This conformational change “increases axonal K+ conductance, which accelerates action potential repolarization, thereby leading to a secondary inhibition of the calcium transient by reducing the degree of calcium channel activation” [22]. GABA amino acid, when bound to the GABA receptor, reduces the frequency of action potentials at nerve cell synapses which reduces neurotransmitter release from the synapse [22]. Thus, GABA B receptors in the nervous system are inhibitory receptors, with inhibitory function when activated by GABA.

Studies indicate that GABA may have a dual action on neurotransmission in the superficial gray layer of the brain. GABA may possess an excitatory effect mediated by GABA A receptors and an inhibitory effect mediated by both GABA A and GABA B receptors [23]. The action of GABA may be dose dependent, inhibitory at low concentrations and excitatory at some GABA receptors at higher concentrations [23]. GABA BRs have been shown to selectively inhibit dendritic L-type Ca^2+^ channels on specific dendrites, leading to reduced calcium influx and loss of long term potentiation at excitatory input synapses onto these interneurons [24].

It has been suggested that the “accumulation of calcium in mitochondria may play a key role as a trigger to mitochondrial pathology. The major process involved is the opening of the mitochondrial permeability transition pore, a large conductance pore that causes a collapse of the mitochondrial membrane potential, leading to ATP depletion and necrotic cell death or to cytochrome c release and apoptosis, depending on the rate of ATP consumption” [25].

Analogously, excitotoxicity of nerve cells is the process characterized by a massive glutamate release in the central nervous system in response to, for example, ischemia, trauma, or substances. Mitochondria accumulate postischemic calcium entering the neurons via the chronically activated N-methyl-D-aspartate receptor [26]. This calcium accumulation plays a key role in the subsequent death of the neuron. “Cultured cerebellar granule cells demonstrate delayed calcium deregulation (DCD) followed by necrosis upon exposure to glutamate” [26].

At least one study has found “that GABA BR … is protective against the NMDA-induced neurotoxicity mediated by [the] mitochondrial [permeability transition pore] PTP through a mechanism related to opening of membrane [G protein-coupled inwardly rectifying K+ ion channels] GIRKs in neurons” [27]. To avoid synaptic hyperexcitation, excitatory output of pyramidal neurons is counterbalanced by input from inhibitory interneurons via binding of the presynaptically released neurotransmitter GABA to the GABA receptor on the postsynaptic membrane [28].

Thus, calcium regulation or inhibition of calcium ion channels is performed by the GABA B receptors through a G protein couple cascade, when activated by GABA or other GABA receptor agonists, including hormones.

### 2.2. Progesterone and *γ*-Aminobutyric Acid

Progesterone is a neurosteroid that acts on GABA receptors via mechanisms described herein. Research has shown that estrogens and progesterone can regulate a number of GABA receptors in rat brain. Both hormones act in selected brain areas. Progesterone, in particular, is a GABA agonist which potentiates the GABA anxiolytic effect. Progesterone has been shown to increase the GABA anxiolytic effect by 160%, and the number of CNS areas responsive to progesterone suggests that sex hormones may play an important role in the regulation of the functions of GABAergic transmission in the central nervous system [29].

Studies of influx demonstrate that progesterone treatment increased the sensitivity of cortical synaptoneurosomes to GABA [30]. The psychotropic effects observed after “progesterone administration are due to the bioconversion of progesterone to allopregnanolone, which subsequently augments GABA A receptor-mediated function” [30].

**Figure 2:**
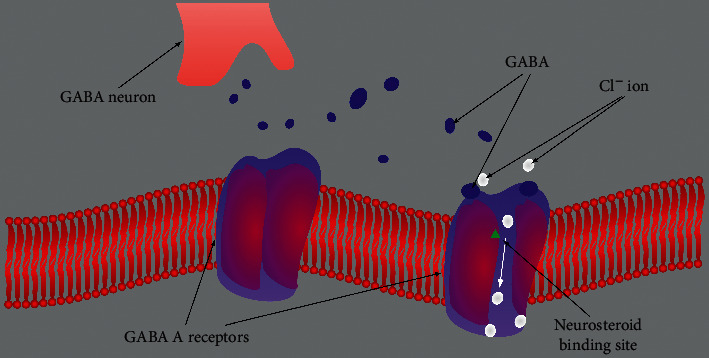
An image of a GABA A receptor, closed left image and open right image to ions, and its GABA and neurosteroid ligand/hormone binding sites. Extrasynaptic GABA A receptors primarily contribute to tonic inhibition. Neurosteroids, including progesterone, allopregnanolone, and DHEA, activate both postsynaptic and extrasynaptic receptors to enhance the phasic and tonic inhibition and thereby promote maximal net inhibition. The blue dots represent the GABA neurotransmitter molecules released from the vesicles of the GABA neuron synapse; the white dots represent chloride ion movement through the GABA A receptor. Abbreviations: GABA, *γ*-aminobutyric acid; DHEA, dehydroepiandrosterone; DHEAS, dehydroepiandrosterone sulfate; CL ion, Cl^−^ion. [35].

“On outside-out membrane patches, 5*β*-pregnan-3a-ol-20-one and 5*β*-pregnane-3,20-dione activated single channel currents of similar amplitude to those evoked by GABA. “The neurosteroid allopregnanolone, allosterically activates the GABA-A receptor … [31]. This modulates the expression and the responsiveness of the GABA-B receptor [31, 32], and in turn its desensitization” Faroni and Magnaghi [33]. The results suggest that certain naturally occurring steroids potentiate the actions of GABA and, additionally, directly activate the GABA A receptor” [34] (see Figure 2). Conversely, the loss of progesterone with menopause can significantly affect the GABAergic system and calcium channel regulation.

### 2.3. DHEA and *γ*-Aminobutyric Acid

DHEA levels start relatively low at birth and gradually increase until puberty, when levels increase markedly, reaching a peak around 20 to 24 years of age. From there, serum and tissue DHEA level decline at a rate of 2 to 3% per year, with a steep decline occurring around middle age [36]. DHEA is a neurosteroid that acts on GABA receptors via mechanisms described herein. One study showed that “alterations in DHEA are involved in the pathophysiology of depression and that the antidepressant action of DHEA is mediated via GABA ARs in the brain tissue” [37]. The mechanism by which DHEA acts as antidepressant is via modulation of the GABA AR activity. The GABAergic system has been shown to mediate depression [38]. Cerebral spinal fluid GABA levels are lower in depressed patients than in healthy controls [39]. Low GABA levels associated with aging are correlated with depression in postmortem temporal and frontal cortices from patients with Alzheimer's disease [37]. Thus, the loss of DHEA hormone associated with aging can affect GABAergic transmission, and calcium channeling, and result in depression.

### 2.4. *γ*-Aminobutyric Acid and Aging

Almost all brain structures, “although anatomically and physiologically diverse (e.g., using different combinations of neurotransmitters), share a common mechanism that controls their activity and metabolism. The control is maintained through a complex interaction between gamma-aminobutyric acid and calcium-dependent neurotransmission and calcium-dependent neuronal metabolism” [40].

“The Ca^2+^/GABA mechanism stabilizes neuronal activity at both the cellular and systemic levels. This close interaction, in addition to the well-documented role of Ca^2+^ in brain aging and neurodegeneration, allows ... [conjecture] that a collapse of the GABA system may be decisive in the initiation and running of these processes.” [40].

In aging, Hua et al. found that the density of GABA-immunoreactive (GABA-IR) neurons was significantly lower in the visual cortex of cats [41]. Studies have also shown loss of inhibitory synaptic contacts in aging.

“Gradual loss of symmetric synapses was confirmed, for example, in layers 2 and 3 of the monkey prefrontal cortex by Peters et al. [42] and decreased density of GABAergic boutons was found in the frontal and parietal cortices of aged rats [43]. Declines in the number and complexity of GABAergic terminals unrelated to neuronal loss were observed in the hippocampus of a mouse model of Alzheimer's disease [44, 45]. Recent studies suggest that inhibitory deficits may also be related to the dysfunction of particular interneuronal subpopulations and their circuits [46]. In accordance with this hypothesis, Akbarian et al. [47] demonstrated reduced expression of [the enzyme glutamate decarboxylase] GAD in the absence of significant cell loss in schizophrenic brains. However, dysfunction and pruning of synaptic endings may occur simultaneously, preceding neuronal loss as observed in Alzheimer disease [48, 49].”

Thus, loss of GABAergic function is associated with aging.

### 2.5. *γ*-Aminobutyric Acid and Brain

**Figure 3:**
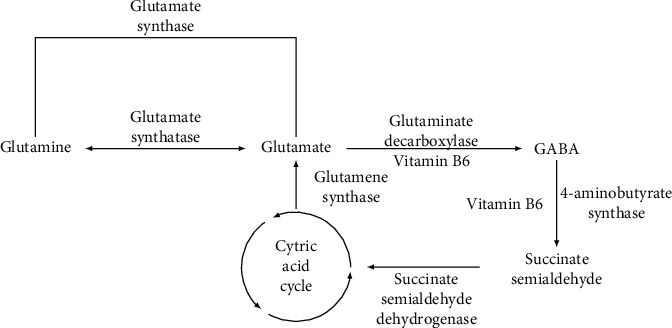
The metabolic pathway of the transformation of glutamate to GABA via the glutamate decarboxylase enzyme and vitamin B6.

Consistent with age-related decreases in GABA inhibitory activity, many studies have actually demonstrated decreases in the synthesis and level of GABA in aging. GABA is synthesized from glutamate by its decarboxylation, catalyzed by the two isoforms of the enzyme glutamate decarboxylase (GAD65 and GAD67) [49] (see also Figure 3). “Many studies have reported aging-associated alterations in the levels of both GAD isoforms in different brain areas. In most cases, the number of GAD IR neurons, the amount of protein, or the mRNA level decreased with age” [49].

Conversely, GABA B receptor activation, such as by GABA administration, promotes nerve regeneration and likely ameliorates neuropathic pain [50]. Sun et al. found that GABA attenuates amyloid toxicity by downregulating amyloid endocytosis and improves cognitive impairment [51].

“GABA downregulated amyloid-*β* (A*β*) uptake in neurons through the receptor for advanced glycation end-products. Therefore, relative[ly] high levels of GABA decreased cytotoxicity induced by A*β* n wild type (WT) mice. GABA treatment decreased basal levels of cell death and the cell death induced by hydrogen peroxide in WT and A*β*PP/PS1 neurons.” [51].

“Recent studies in the mouse brain and pancreas suggest GABA might also play an important role in regeneration distinct from its neurotransmitter role. Scientists studying the hippocampus region of the brain found that GABA levels regulate the activity of neural stem cells. When GABA levels are high, the stem cells stay quiet, and if GABA levels decrease, then the stem cells start to divide.” [52].

Studies have shown loss of GABAergic function with aging and that GABA treatments may hold promise in addressing GABAergic dysfunction associated with aging.

### 2.6. *γ*-Aminobutyric Acid and Sleep

There is evidence for a physiological modulation of GABAergic activity by melatonin [53]. In addition, GABA administration can improve sleep disturbances that are associated with aging [54]. In particular, a “GABA/l-theanine mixture led to a significant increase in rapid eye movement (REM) (99.6%) and non-REM (NREM) (20.6%) sleep compared to controls. The use of GABA/l-theanine mixture rather than GABA or l-theanine alone restored to normal levels sleep time and quality in the arousal animal model” [54]. Theanine and GABA in tea have been found to lower cortisol levels [55]. Addressing GABAergic dysfunction associated with aging can improve sleep.

### 2.7. *γ*-Aminobutyric Acid and Inflammaging

GABA also influences the immune system in aging. Inflammaging is defined as low-grade chronic systemic inflammation established during physiological aging. A steady decline in the production of fresh naïve T cells, more restricted T cell receptor (TCR) repertoire, and weak activation of T cell are some of the effects of aging [56].

“There is evidence that certain types of high voltage activated calcium channels are involved in neurogenic inflammation and inflammatory pain, in agreement with reports indicating the therapeutic effectiveness of gabapentinoids [modulators of GAD], and ligands for the *α*2*δ* subunit of high voltage activated channels, in treating not only neuropathic, but also inflammatory, pain.” [57].

In responder T cells, GABA decreased proliferation and inhibited secretion of 37 cytokines in a concentration-dependent manner. In the nonresponder T cells, GABA modulated release of 8 cytokines. GABA amino acid concentrations in plasma from type 1 diabetes (T1D) patients and ND individuals were correlated with 10 cytokines where 7 were increased in plasma of T1D patients. GABA inhibited secretion of 5 of these cytokines from both T1D PBMCs and ND responder T cells. The results identify GABA as a potent regulator of Th1- and Th2-type cytokine secretion from human PBMCs and CD4+ T cells where GABA generally decreases the secretion [58].

IgE-mediated histamine release is also suppressed by GABA, with about 60% inhibition at 100 microM, in rat studies [59].

GABA affects the immune system and decreases inflammatory cytokines and histamine levels. Loss of GABA and GABAergic function with aging can contribute to inflammaging.

### 2.8. *γ*-Aminobutyric Acid and Autophagy

Autophagy is the natural, regulated mechanism of the cell that removes unnecessary or dysfunctional components from the body. It allows for the orderly degradation and recycling of cellular components. “Treatment of macrophages with GABA or GABAergic drugs promotes autophagy activation, enhances phagosomal maturation and antimicrobial responses against mycobacterial infection. In macrophages, the GABAergic defence is mediated via a macrophage type A GABA receptor, intracellular calcium release” [60].

Deficits in GABA inhibition and autophagy have been associated with accumulation of cell products, including amyloid and other proteins in the brain. Recent evidence suggests that impaired autophagy is one mechanism that influences the clearance of protein aggregates and neurodegenerative disease pathogenesis [61–63]. Therefore, loss of GABAergic transmission with aging can affect the immune system and levels of autophagy and allow for accumulation of protein aggregates in body systems, including the brain.

### 2.9. *γ*-Aminobutyric Acid and Blood Pressure

With respect to cardiovascular control, GABA has been known to have an effect on blood pressure since 1954 [64]. Studies suggest an important role for GABA receptors in the central regulation of blood pressure and heart rate [65]. Baroreceptors are mechanoreceptors located in the carotid sinus and in the aortic arch. Their function is to sense pressure changes by responding to change in the tension of the arterial wall. The baroreflex mechanism is a fast response to changes in blood pressure [66]. GABA mediates baroreceptor inhibition of reticulospinal neurons. In one study, “every reticulospinal neuron unit tested was inhibited by iontophoretic applications of gamma-aminobutyric acid (GABA)” [67]. Thus, one way in which GABA affects blood pressure has been elucidated, through baroreceptors.

In another study of blood pressure (BP) control with GABA, subjects took 80 mg of GABA daily and saw that systolic BP and diastolic BP measured in the morning were significantly reduced by 10 and 5 mmHg (*p* < 0.05) compared to baseline BP in the GABA treatment group [68]. GABA is also diuretic [69].

Therefore, changes in the GABAergic system and loss of function of GABA at synapses with aging can cause increases in blood pressure and failure of baroreceptors to function properly.

### 2.10. *γ*-Aminobutyric Acid, Heart, and Blood

The sinoatrial node is the natural pacemaker of the heart that determines heart rate in mammals, including humans. It is characterized by the ability to generate spontaneous action potentials or changes in membrane potential between the outside and the inside of the cell that serve to excite the surrounding atrial myocardium. It has been proposed that GABA modulates neurotransmission in the sinoatrial node of the heart via stimulation of the GABA A receptor, which affects heart rhythm [70]. GABA levels in circulation also promote blood cell maintenance. One study identified a conserved link between the neural product GABA and hematopoietic systems in mice and humans that may provide a strategy for producing megakaryocyte progenitors and then platelets by manipulating GABA R R1 (GABA receptor rho subunit) mediated GABA signaling [71]. In the study, “GABA antagonist treatment inhibited megakaryocyte and platelet differentiation, while activation of GABA R R1 through lentivirus-mediated overexpression or GABA agonist treatment promoted platelet generation” [71]. Thus, it is likely that adequate GABA levels support heart pace making and blood maintenance in aging.

### 2.11. *γ*-Aminobutyric Acid and Vision

Kurcyus et al. investigated levels of GABA and glutamate “in the visual cortex of healthy human participants (both genders) in three functional states with increasing visual input. Compared with a baseline state of eyes closed, GABA levels decreased after opening the eyes in darkness and Glx levels (Glutamate complex Glx, is a complex of Glu and glutamine [Gln]) remained stable during eyes open but increased with visual stimulation” [72]. Other studies have discovered that “decreased levels of gamma-aminobutyric acid (GABA) cue the retina to produce stem cells in zebrafish, shedding light on how zebrafish regenerate their retina after injury and informing efforts to restore vision in blind people” [73]. Thus, emerging research shows that GABA is involved in vision signaling. It will be an interesting area for further research to determine whether support of the GABAergic system supports healthy vision aging.

### 2.12. *γ*-Aminobutyric Acid and Bone

Studies have shown “the local production of *γ*-aminobutyrate (GABA) in hypertrophic-zone chondrocytes of the rat tibial growth plate, an important long bone growth site” [74]. In addition, GABA contributes to a mouse chondrogenic cell line proliferation via GABA A and GABA B receptors and these mechanisms may also be involved in cartilaginous cell growth [74]. While further human studies are necessary, GABA may also support bone and cartilage maintenance in human aging. However, GABAergic homeostasis is a delicate balance. GABA acts as a neurotrophic factor during nervous system development and has been found to be related to the proliferation and migration of certain bone cancer cells. At least one study found that the GABA B receptor antagonist compound CGP had antitumor effects on high-grade chondrosarcoma cells in a dose-dependent manner through cell cycle arrest and induced apoptosis. Changes in intracellular Ca^2+^ via GABA B receptor-related Ca^2+^ channels inhibited the proliferation of high-grade chondrosarcoma cells by inducing and modulating apoptotic pathways [75].

### 2.13. *γ*-Aminobutyric Acid and Lungs

It has been shown that activation of GABA receptors causes respiratory depression. This suggests that “GABA may be an important neurotransmitter in CNS neural pathways involved in regulating respiratory activity” [76]. Therefore, alterations in the GABAergic system with aging can likely affect respiratory function.

### 2.14. *γ*-Aminobutyric Acid and Kidney Function

A kidney function study demonstrated that GABA attenuated renal dysfunction via “regulation of blood pressure and lipid profile, and it also ameliorated the oxidative stress induced by nephrectomy, suggesting the promising potential of GABA in protecting against renal failure progression” [77]. Changes to the GABAergic system with aging can affect kidney function.

### 2.15. *γ*-Aminobutyric Acid and Thyroid Function

GABA also regulates thyroid function, which can decrease with aging. Different doses of GABA increased thyroxine (T4), triiodothyronine (T3), and thyroid hormone receptor beta (TH*β*) to levels significantly higher than those in a NCG (negative control group) [78].

“The effect of GABA on increasing T4 was inversely related to the dose; thus, the effects of GABA decreased T4 with increasing dosage. In a PCG (positive control group), thyroid tablets had positive effects on T4, T3 and TH*β* levels. The T4 and T3 levels in PCG and GABA treatment groups remained significantly lower than those of the control group (*p* < 0.05).” [78].

Thus, the presence of GABA in this study improved thyroid function by keeping thyroid hormone at appropriate levels and preventing thyroid hormone dysfunction.

It has also been demonstrated that “the addition of 50–100 mg/kg GABA to the diet of chickens under heat stress significantly increased T3 blood concentrations. Moreover, Xie et al. [79] demonstrated that treatment with 0.2% and 0.12% GABA normalized plasma TSH levels, improved plasma T4 levels and normalized the morphology of follicles in high-fat-diet-fed mice” [78]. Treatment of fluoride-exposed mice with GABA significantly decreased the metabolic toxicity induced by fluoride and restored the microstructural and ultrastructural organization of the thyroid gland [78].

Therefore, GABA administration may assist thyroid function and prevent thyroid gland toxicity induced by fluoride in the environment.

### 2.16. *γ*-Aminobutyric Acid, Growth Hormone, and Muscle

GABA seems to directly stimulate growth hormone GH secretion via centrally mediated mechanisms. GABA administration has been shown to stimulate GH secretion at rest [80, 81]. A Japanese study found that “daily supplementation using GABA and whey protein significantly enhanced whole body fat-free mass after 12 weeks of resistance training among untrained middle-aged men with suboptimal dietary protein intake, which suggests that GABA (in combination with whey protein) may help enhance exercise-induced muscle hypertrophy. GABA's ability to enhance muscle hypertrophy may be related in part to its ability to increase basal GH concentrations. Oral GABA supplementation promotes protein synthesis in various parts (e.g. the brain and gastrocnemius muscle) and higher plasma GH concentrations at rest in rats” [81].

GABA may assist muscle maintenance in aging. Additionally, “[c]hanges to inhibitory signaling by GABA are thought to be crucial in inducing motor cortex plasticity” [82]. Therefore, differing GABA levels appear to be critical for muscle growth and muscle learning which may be considerations for the aged.

### 2.17. *γ*-Aminobutyric Acid and Hearing

GABA is critical to maintaining hearing as you age. “Ling et al. [83] reported reduced levels of GAD65 and GAD67 mRNAs, and Burianova et al. [84] reported an age-related decrease in the protein levels of GAD65 and GAD67 in the rat auditory cortex. They suggested that the observed changes may contribute significantly to the deterioration of hearing function” [49]. Experiments have shown that “tinnitus, which often increases with aging, is correlated with lower levels of the inhibitory neurotransmitter GABA, but not with changes in the excitatory neurotransmitters. It has been demonstrated that two drugs that increase the level of GABA eliminated tinnitus in rats” [85].

Thus, GABAergic dysfunction in aging can affect hearing.

### 2.18. *γ*-Aminobutyric Acid and Cancer

GABA has been found to increase some tumor growth types and to decrease other tumor growth types. “GABA was found to play an inhibitory role in colon carcinoma [86, 87], cholangiocarcinoma [88], and lung adenocarcinoma [89]. The activation of GABA B receptors has an inhibitory effect on most of human tumor types, except prostate cancer [90]” [91].

Therefore, the loss of GABAergic function with aging may play a role in cancer development.

### 2.19. *γ*-Aminobutyric Acid, Pancreatic Function, and Metabolic Syndrome

GABA is a major neurotransmitter in the central nervous system (CNS), where GABA produces fast inhibition in mature neurons primarily by activation of A-type GABA receptor (GABA AR).

“A large amount of GABA is also produced in the pancreatic islet [92], where it exists at the highest concentration outside of the CNS [93]. Pancreatic GABA is primarily produced by the *β*-cell [94]. In the *β*-cell, GABA is stored in synaptic-like microvesicles that are distinct from insulin-containing large-dense core vesicles [95]. However, recent evidence indicates that GABA is co-localized with insulin in core vesicles in human islets and that the release of GABA from the *β*-cells is glucose-dependent [96]. The release of GABA from *β*-cells is “tonic” [97, 98], yet the amount of released GABA is regulated by the metabolic state of *β*-cells [99, 100].”

In the pancreatic islet, GABA released from *β*-cells plays an important role in the regulation of glucagon secretion from *α*-cells. Specifically, GABA activates GABA ARs in *α*-cells of the pancreas, with hyperpolarization leading to an inhibition of glucagon secretion, analogous to how GABA synapses work in the brain [101].

“The GABA AR-mediated hyperpolarization of *α*-cells represents a physiological mechanism for glucose-induced suppression of glucagon release because blockade of the GABA AR has been shown to diminish the inhibitory effect of high glucose on glucagon secretion in isolated rat [102] or mouse [103] islets. It has been demonstrated that insulin suppresses glucagon secretion by enhancing intra-islet GABA-GABA AR signaling through translocation of GABA AR from an intracellular pool to the cell surface of *α*-cells [101, 104].”

Excessive secretion of glucagon is a major contributor to the development of diabetic hyperglycemia. Secretion of glucagon from the pancreas signals the liver to convert stored glycogen to glucose. This process is regulated by various nutrients, with glucose being a primary determinant of the rate of pancreatic *α* cell glucagon secretion. The intraislet action of insulin is essential to exert the effect of glucose on the pancreatic *α* cells. In the absence of insulin, glucose is not able to suppress glucagon release in vivo. A recent study shows that “insulin induces activation of GABA A receptors in the *α* cells by receptor translocation. This leads to membrane hyperpolarization in the pancreatic *α* cells and, ultimately, suppression of glucagon secretion. Defects in this pathway(s) contribute to diabetic hyperglycemia” [101].

In order to investigate a possible role of GABA in the regulation of insulin secretion,

“researchers studied the effect of GABA on insulin secretion from the isolated perfused rat pancreas in vitro and on the changes in the cytoplasmic Ca^2+^ of Beta-cells from the isolated rat islets. When glucose is present, GABA caused a dose dependent inhibition of the first phase of arginine-induced insulin secretion during the range of 10–1000 *μ*M, but GABA did not affect arginine-induced insulin secretion in the absence of glucose. GABA inhibited not only the first phase but also the second phase of glucose-induced insulin secretion. A GABA B-receptor agonist, baclofen, also inhibited both phases of insulin secretion induced by 16.7 mM glucose. Furthermore, GABA inhibited the rise in cytoplasmic Ca^2+^ of Beta-cells in response to 16.7 mM glucose. These studies indicate that GABA decreases Beta cell secretory activity mainly in response to glucose. These inhibitory effects of GABA on insulin secretion may be mediated through GABA B-receptor and the inhibition of the rise in cytoplasmic Ca^2+^” [105].

These studies found that “if the delivery of insulin or GABA plus insulin in rats with hypoglycemia is terminated, *β*-cells are stimulated and signal the *α*-cells to secrete glucagon. Thus, the detection of a sudden decrease in zinc levels by *β*-cells as well as a decrease in GABA in ... circulation induces signaling to *α*-cells to stimulate them to secrete glucagon” [106]. These studies also show that GABA decreases beta cell secretory activity mainly in response to glucose.

Importantly, recent studies performed in rodents, including in vivo studies of transplanted human pancreatic islets, reveal that GABA exerts pancreatic *β*-cell regenerative effects. Moreover, GABA protects *β*-cells against apoptosis induced by cytokines, drugs, and other stresses and has anti-inflammatory and immunoregulatory activities. It ameliorates the manifestations of diabetes in preclinical models, suggesting potential applications for the treatment of diabetic patients [107].

It has been also shown that “GABA can prevent and reverse the development of diabetes in type 1 mice models” [108]. In addition, “GABA appears to be beneficial to T2D. Tian et al, [109] demonstrated that oral treatment with GABA improves glucose tolerance and insulin sensitivity in high-fat diet-fed mice. They concluded that this was due to the inhibition of obesity-related inflammation and upregulation of Treg responses” [107].

Treatment of mice with “GABA ... leads to massive neogenesis with the formation of more and larger islets. The drugs appear to activate the pancreatic duct epithelium. This is associated with increased proliferation of epithelial cells as glucagon-expressing cells develop into new *β*-like cells, which are hyperplastic and form new islets” [110]. “GABA increases the mitotic rate of *β* cells. In mice, following *β*-cell depletion with streptozotocin, GABA therapy can restore the *β*-cell mass” [111].

Furthermore, oral treatment with GABA significantly reduced the concentrations of fasting blood glucose and improved glucose tolerance and insulin sensitivity in the high-fat-diet- (HFD-) fed mice. More importantly, after the onset of obesity and type 2 diabetes mellitus (T2DM), oral treatment with GABA inhibited the continual HFD-induced gain in body weights, reduced the concentrations of fasting blood glucose, and improved glucose tolerance and insulin sensitivity in mice. In addition, oral treatment with GABA reduced the epididymal fat mass, adipocyte size, and the frequency of macrophage infiltrates in the adipose tissues of HFD-fed mice. Collectively, these data indicated that activation of peripheral GABA receptors inhibited the HFD-induced glucose intolerance, insulin resistance, and obesity by inhibiting obesity-related inflammation and upregulating Treg responses in vivo. Given that GABA is safe for human consumption, activators of GABA receptors may be valuable for the prevention of obesity and intervention of T2DM in the clinic [109].

GABA is essential for pancreatic function. Loss of GABAergic function with aging can cause dysregulation of pancreatic function and result in changes in insulin and blood sugar levels. New research shows that restoration of some pancreatic islet function is possible with administration of GABA.

### 2.20. *γ*-Aminobutyric Acid and Liver Function

A study revealed that a complex GABA signaling system exists in the rat liver. Activation of this intrahepatic GABAergic system protected the liver against toxic injury [112].

GABA plus caloric restriction showed reduced fasting blood glucose (FBG) levels and improved whole body insulin responsiveness indicated by increased insulin levels, insulin sensitivity, and glucose tolerance. This combination therapy improved the lipid profile by significantly reducing triglycerides, total cholesterol, and LDL levels and increased HDL levels. The transcript expression profile of metabolic enzymes in the liver indicates a significant decrease in gluconeogenesis and glycogenolysis with this therapy. The transcript levels indicate decreased lipolysis and increased fatty acid synthesis in the adipose tissue. The transcript levels of mitochondrial biogenesis in the skeletal muscle show an increased effect from the combination of GABA and caloric restriction and improved insulin sensitivity. Also, this combination therapy promoted *β*-cell proliferation. In conclusion, calorie restriction in combination with GABA ameliorates T2D in the mouse model by inducing *β*-cell regeneration mediated by GABA and increased insulin sensitivity by caloric restriction (CR) diet [113]. GABA is protective of liver function, which may be a consideration for the aging population.

### 2.21. *γ*-Aminobutyric Acid and Weight

Similarly, dietary GABA decreases body weight of genetically obese mice.

“Body weight and food intake were decreased more in lean than in genetically obese ... mice fed a low protein diet containing GABA. Young lean and obese mice, when fed a 4.5% GABA diet, lost 30% or 20% of their initial weight over periods of 10 or 32 days, respectively. With GABA at 2 or 2.5% of the diet, respectively, lean and obese mice could be maintained at constant low weights for weeks.” [114].

Therefore, while human studies are necessary, it may be possible that some of the weight gain associated with aging may be attributed to loss of GABAergic function and can potentially be addressed with administration of GABA.

### 2.22. Skin, Joints, and *γ*-Aminobutyric Acid

Researchers examined the effects of GABA on type I collagen gene expression in normal human dermal fibroblasts. “Real-time PCR analysis indicated GABA increased the level of type I collagen transcripts, and suppressed the expression of matrix metalloproteinase-1, which is a collagen-degrading enzyme. These results suggest GABA improves the skin elasticity by regulating type I collagen expression” [115].

Researchers have also shown that “activation of peripheral GABA-Rs can inhibit the development of disease in the collagen-induced arthritis (CIA) mouse model of RA. Mice that received oral GABA had a reduced incidence of CIA, and those mice that did develop CIA had milder symptoms” [109].

Therefore, GABA may have potential to reduce the incidence of collagen-induced arthritis associated with aging.

### 2.23. *γ*-Aminobutyric Acid and Pain

Old age is associated with aches and pains. It is known that the “[c]urrent understanding of chronic pain points to a decrease in level of the inhibitory neurotransmitter GABA, in the spinal dorsal horn, leading to an imbalance between excitatory and inhibitory pathways” [116]. A 2010 study found that special “GABA cells can modulate neuropathic pain ... by minimizing the imbalance and restoring the cellular GABAergic pathway” [116].

Therefore, addressing and supporting the GABAergic system may help with the aches and pains of old age.

### 2.24. *γ*-Aminobutyric Acid, the Food Supply, and Nutrition

#### 2.24.1. Glyphosate

Research on animal models has confirmed the neurotoxic effects of glyphosate. For example, Negga et al. found that exposure to glyphosate causes neuronal death, especially of GABAergic and dopaminergic neurons in the nematode *Caenorhabditis elegans* [117]. “Glyphosate is neurotoxic [118]. Its mammalian metabolism yields glyoxylate. Glyoxylate is a highly reactive glycating agent, which will disrupt the function of multiple proteins in cells that are exposed [119, 120].” An investigation of glyphosate's effects on oxidative stress parameters, as well as glutamate uptake, release, and metabolism was conducted.

“Results showed that acute exposure to Roundup (®) (30 min) increases (45) Ca (2+) influx by activating N-methyl-D-aspartate (NMDA) receptors and voltage-dependent Ca (2+) channels, leading to oxidative stress and neural cell death. Acute exposure to Roundup (®) increased (3)H-glutamate released into the synaptic cleft, decreased GSH [glutathione] content and increased the lipoperoxidation, characterizing excitotoxicity and oxidative damage. These results demonstrated that Roundup (®) might lead to excessive extracellular glutamate levels and consequently to glutamate excitotoxicity and oxidative stress in rat hippocampus.” [118].

Thus, glyphosate has been shown to cause a disruption of calcium signaling, a decrease in GABAergic function, and glutamate/GABA imbalance.

Data suggest that exposure to TD and/or MZ (TouchDown(®) (TD) active ingredient (glyphosate), or Mancozeb(®) (MZ)) promotes neurodegeneration in both GABAergic and dopaminergic neurons in the model organism *C. elegans* [121]. Thus, the combination of the loss of GABAergic function associated with aging and the presence of toxic chemicals like glyphosate in the food supply, or fluoride in the water supply, leads to further stress on the GABAergic system.

#### 2.24.2. Monosodium Glutamate (MSG)

Monosodium glutamate is an excitotoxin of the GABAergic system [122]. Excess glutamate allows high levels of calcium ions (Ca^2+^) to enter the cell. Ca^2+^ influx into cells activates a number of enzymes, including phospholipases, endonucleases, and proteases such as calpain. These enzymes go on to damage cell structures such as components of the cytoskeleton, membrane, and DNA [123]. Experimental studies have also shown that “the subcutaneous injection of 2 mg/g body weight monosodium glutamate (MSG) into newborn mice results in central obesity and moderate to severe microvesicular fatty changes throughout the liver parenchyma at 6-months age. In this animal model, the administration of MSG causes an increase in fasting blood glucose levels and ultimately T2DM. The higher doses of parenteral MSG (4 mg/g body weight) cause insulin resistance (IR) in mice evidenced by the significant increase in plasma glucose in oral glucose tolerance test and severe visceral fat accumulation. Histopathological examination of these models showed pancreatic islets hypertrophy, hyperplasia and decreased pancreatic *α*-cells. The toxic effects of MSG on the CNS in animal studies is not entirely applied to the human MSG intake because it depends on some factors, such as, the age, route of administration and the dose” [124].

MSG, in a manner similar to glyphosate, causes excitotoxicity of the GABAergic system and affects the GABAergic system negatively. MSG should be avoided in everyone, particularly in the diet of the aged.

#### 2.24.3. Aluminum and *γ*-Aminobutyric Acid

Researchers have found that aluminum is toxic to the GABAergic system. Aluminum caused a marked increase in glutamate and glutamine levels while GABA levels were significantly decreased [125]. Therefore, the presence of aluminum in makeup, deodorants, cooking utensils, etc. could lead to an imbalance in glutamate and GABA levels, tending towards excitotoxicity.

#### 2.24.4. Dietary Fructose and *γ*-Aminobutyric Acid

An excellent study by Hassel et al. measured metabolism of [14C]glucose and [14C]fructose by isolated nerve endings. Isolated nerve terminals (synaptosomes) were prepared from neocortex of Wistar rats and incubated in a Krebs solution containing 1 *μ*Ci [U-14C] glucose and [U-14C] fructose [126]. Glucose metabolism in nerve cells produced about half of the glutamate compared to the amount of glutamate produced by the nerve cell metabolism of fructose. Metabolism of fructose in the nerve cells led to a significantly increased production of glutamate as compared to GABA and as compared to glucose metabolic products.

Therefore, dietary fructose may contribute to high glutamate production and decreased GABA production, contributing to GABAergic imbalance and an excitotoxic state.

#### 2.24.5. *γ*-Aminobutyric Acid in Food

GABA has emerged as a promising compound that is able to regulate cancer by the “induction of apoptosis and inhibition of proliferation and metastasis ... Gaba-enriched brown rice extract significantly retarded the proliferation rates of L1210 and Molt4 leukemia cells and enhanced apoptosis of the cultured cancer cells. Moreover, Schuller et al. suggested that GABA had a tumor suppressor function in small airway epithelia and pulmonary adenocarcinoma” [16].

“Food technologies and molecular engineering [have been] employed to synthesize GABA through enzymatic or whole-cell biocatalysis, microbial fermentation (for example, GABA soya yogurt …, black raspberry juice … ), and chemical synthesis …. Some authors found one of the highest contents on GABA to be 414 nmol/g of dry weight in raw spinach, followed by Solanum tuberosum L. (that is, potato), Ipomoea batatas L. (that is, sweet potato), and Brassica oleracea L. (that is, cruciferous such as kale and broccoli). Mushrooms, such as Lentinula edodes B. (that is, shiitake), and nuts of Castanea genus (that is, chestnut) also showed a significant amount of GABA …. Among the many types of Chinese teas, the highest GABA content was found in white tea …. As already mentioned, GABA content was found in mistletoe …, but also in Phytolacca americana L. (that is, pokeroot) …, Valeriana officinalis L. (that is, valerian), Angelica archangelica L. (that is, wild celery), Hypericum perforatum L. (that is, St John's wort), Hieracium pilosella L. (that is, mouse-ear hawkweed), and Passiflora incarnata L. (that is, maypop) …, the latter being used for the relief of mild symptoms of mental stress and as a sleep aid” (internal citations omitted) [127].

Both the Earth's flora and new food technologies have the potential to provide supportive dietary GABA.

#### 2.24.6. *γ*-Aminobutyric Acid and Probiotics in Diet

Probiotics synthesize GABA and indirectly support GABA production through the function of and production of the glutamate acid decarboxylase (GAD) enzyme (see Figure 3). GAD is an enzyme that catalyzes the decarboxylation of glutamate to GABA and CO_2_ and maintains the major physiological supply of GABA amino acid in mammals. In one investigation “the blood-pressure-lowering effects of *γ*-aminobutyric acid (GABA) and a GABA-enriched fermented milk product (FMG) by low-dose oral administration to spontaneously hypertensive (SHR) and normotensive Wistar–Kyoto (WKY) rats [was tested]. FMG is a nonfat fermented milk product produced by lactic acid bacteria, and the GABA contained in FMG was made from the protein of the milk during fermentation. A single oral dose of GABA or FMG (5 ml/kg; 0.5mgGABA/kg) significantly (*p* > 0.05) decreased the blood pressure of SHR from 4 to 8 h after administration, but did not increase that of WKY rats. The hypotensive activity of GABA was dose-dependent from 0.05 to 5.00 mg/kg in SHR. During the chronic administration of experimental diets to SHR, a significantly slower increase in blood pressure with respect to the control group was observed at 1 or 2 weeks after the start of feeding with the GABA or FMG diet respectively (*p* > 0.05) and this difference was maintained throughout the period of feeding. The time profile of blood-pressure change due to administration of FMG was similar to that of GABA” [128].

These results suggest that a low-dose oral GABA product produced by lactic acid bacteria has a hypotensive effect in SHR and that the hypotensive effect of FMG is due to GABA.

“The genetics of GABA have been elucidated in Escherichia coli …, Lactococcus lactis subsp. Lactis …, and L. brevis …. Sanders et al. … sequenced the L. lactis subsp. lactis gadCB gene and suggested that it encoded a glutamate-dependent acid resistance mechanism comprised of glutamate-GABA antiporter and GAD. Nomura et al. … indicated that L. lactis subsp. Lactis contains a single GAD gene (gadB), while the gram-negative E. coli ... and Shigella sp. … contain two GAD genes. The functional properties of the two E. coli isozymes were identical …. The partial GAD sequences found in this study showed high identity with gadB sequences from L. plantarum WCFS1... , L. brevis ATCC 367 ... , L. lactis subsp. Lactis Il1403 ... ), Listeria monocytogenes strain F6854 ... , Enterococcus faecium DO ... , Lactobacillus reuteri 100-23 ... , Clostridium perfringens strain 13 ... , and E. coli CFT073” (internal citations omitted) [129].

Probiotics work to support good health by supplementing the GABAergic pathway, GAD enzymes, and supplying GABA.

#### 2.24.7. *γ*-Aminobutyric Acid and Soy

Germinated grains have been known as sources of GABA that provide beneficial effects for human health. The highest GABA content has been found in germinated mung bean, followed by germinated soybean, germinated black (soy) bean, and soaked sesame. Studies confirmed that germinated mung bean is a rich source of GABA and dietary fibers. Microwave cooking resulted in the smallest loss of GABA in mung bean and sesame, while steaming led to the least GABA content loss in soybean and black bean [130].

The GABA content of brown rice and mung bean reminds us of the macrobiotic anticancer diet that recommends these foods in the diet to suppress cancer [131].

#### 2.24.8. *γ*-Aminobutyric Acid and Caffeine

Caffeine inhibits GABA release.

“The less GABA, the more nerve transmissions occur. Think what too much coffee feels like: that is the sensation of glutamate without enough GABA. The reason caffeine does this is that other molecules can bind to the neuron near the GABA binding site and influence GABA's effect. Caffeine has been found to suppress the inhibitory (GABAergic) activity and modulate GABA receptors” [132].

In other words, when caffeine is present, GABA is unable to exert its inhibitory effect on GABA receptors, and an excitatory nerve state is present. “Postsynaptically, independently of [Ca^2+^], caffeine competitively binds to multiple regulatory sites of the GABA A receptor and interferes with GABAergic transmission” [133]. “Caffeine also disrupts chloride transporters and shifts the chloride equilibrium potential towards the reduction of its conductance” [134].

Therefore, caffeine negatively affects GABAergic inhibitory function. Thus, caffeine should be used only in moderation as you age to prevent further disruption of the GABAergic systems discussed here.

### 2.25. GABA Supplement

“GABA is rapidly absorbed, with maximum plasma concentrations achieved approximately 1–1.5 h after an oral dose, and subsequent mean elimination half-life in a range of 5–5.2 h” [135]. GABA is rapidly absorbed by the gastrointestinal tract and remains elevated in the circulation for hours; however, GABA does not readily cross the blood brain barrier. For example, *GABA* may be taken in *doses* as small as 100 mg daily, up to 750 mg 2-3 times per day [136]. It may be a beneficial area of further research to provide readily absorbable GABA formulations that cross the blood brain barrier, for obvious reasons.

#### 2.25.1. Vitamin D and *γ*-Aminobutyric Acid

Using an animal model, researchers “characterized the dysfunction of excitatory and inhibitory neurotransmission under alimentary vitamin D (VDD). The shift between unstimulated and evoked GABA release under VDD was largely reversed after treatment of VDD, whereas the impairments in the glutamatergic system were only partially recovered after 1-month vitamin D3 supplementation” [137].

Thus, vitamin D can partially recover impairments in the GABAergic system. The aged should ensure proper levels of vitamin D to support the GABAergic systems discussed here.

#### 2.25.2. *γ*-Aminobutyric Acid and Magnesium

Magnesium has been shown to modulate GABA activity in the brain. Magnesium ions can occupy GABA receptors acting as GABA receptor agonists to help facilitate GABA neurotransmission. GABA is an inhibitory neurotransmitter in the brain that plays a role in motor control, vision, and anxiety [138]. Thus, magnesium can activate inhibitory GABA receptors in the same way GABA does, and magnesium may be an essential supplement for the aged.

#### 2.25.3. Curcumin and *γ*-Aminobutyric Acid

Inhibitory GABA A receptors play a pivotal role in orchestrating various brain functions and represent an important molecular target in neurological and psychiatric diseases, necessitating the need for the discovery and development of novel modulators. One study showed that a natural compound, curcumol, from turmeric, acts as an allosteric enhancer of GABA ARs. Curcumol markedly facilitated GABA-activated (inhibitory) currents [139].

#### 2.25.4. Calcium and *γ*-Aminobutyric Acid

Calcium supplementation appears, under some circumstances, to enhance the activity of GABA receptors. In one study, the effects of different allosteric modulators on the functional activity of gamma-aminobutyric acid (GABA) B receptors were studied using membranes of postmortem human frontal cortex. Ca^2+^ ion (1 mM) enhanced the potency of the GABA receptor agonists GABA and 3-aminopropylphosphinic acid (3-APA), but not the activity of the GABA(B) receptor agonist (-)-baclofen. Likewise, chelation of Ca^2+^ ion by EGTA reduced the positive allosteric modulator of GABA(B) receptors, CGP7930 ((2,6-di-t-Bu-4-(3-hydroxy-2,2-dimethyl-propyl)-phenol), enhancing GABA potency in stimulating binding by twofold [140].

The enhancement of GABA receptor function is consistent with studies finding that oral calcium supplementation augments baroreceptor reflex function, in part through an enhancement of parasympathetic nervous activity, resulting in reduction of the lability of blood pressure in patients with mild to moderate essential hypertension [141] (also, [142]).

#### 2.25.5. Zinc and *γ*-Aminobutyric Acid

Zinc ions are concentrated in the central nervous system and regulate GABA(A) receptors, which are pivotal mediators of inhibitory synaptic neurotransmission. Zinc ions inhibit GABA(A) receptor function by an allosteric mechanism that is critically dependent on the receptor subunit composition [143]. Zinc exerts a tonic inhibition of GABA A receptors.

#### 2.25.6. Vitamin B6 and *γ*-Aminobutyric Acid

The vitamin B6 vitamers include pyridoxine, pyridoxal, and pyridoxamine, as well as their phosphorylated forms such as pyridoxal phosphate, which are a key coenzyme for a large variety of enzymes involved in many aspects of metabolism. GAD (glutamic acid decarboxylase) is a PLP (pyridoxal-5-phosphate) dependent enzyme that catalyzes the *α*-decarboxylation of l-glutamate into the essential mammalian inhibitory neurotransmitter GABA (*γ*-aminobutyric acid) [144].

Figure 3 shows the metabolic pathway of the transformation of glutamate to GABA via the glutamate decarboxylase enzyme and vitamin B6 [145].

Vitamin B6 also contributes to the synthesis of many neurotransmitters [146]. A study was undertaken to investigate the effects of vitamin B6 administration on age-related changes in rat brain. The animals were injected intraperitoneally with 1, 10, and 100 mg vitamin B6/kg body weight/day for 30 days, and specific activity of GAD was assayed in the brain supernatant [147]. The activity of the enzyme in aged rats was significantly lower as compared to that of young animals. Vitamin B6 induced activation of the brain enzyme in both ages, but the rate of the activation was markedly pronounced in aged animals. The authors concluded that the “[s]ignificant activation rate of GAD by vitamin B6 in aged rat brain may be resulted from either lower availability of vitamin B6 in aged animals, or lower affinity of the enzyme for pyridoxal-5-phosphate, which was determined to be related to conformational changes of the enzyme during aging. It is suggested that vitamin B6 may restore the activity of the brain glutamate decarboxylase in aged rat” [147].

Supplementation with vitamin B6, and/or its metabolites, in the aged may support the aging GABAergic system.

#### 2.25.7. Exercise and *γ*-Aminobutyric Acid

Exercise is important for maintaining a healthy GABAergic system in the aged. For example, researchers studied the effect of exercise on GABA signaling pathways in a model of chemically induced seizures in rats. Exercise was found to reduce the severity and increase the latency of seizures in the seizure exercise rat group. The gene level of GAD65 and GABA A receptor *α*1 was highly expressed in the seizure exercise rate group in the hippocampal area. GABA A receptor *α*1 was upregulated in several areas of the brain in the exercise group [148].

Other studies have demonstrated that exercise can upregulate GABA-mediated caudal hypothalamic control of cardiovascular function in SHR (spontaneously hypertensive rats). Similarly, exercise increases GAD gene transcript levels in the posterior hypothalamus. Thus, researchers have identified a model to study exercise-related central neural plasticity in GABAergic neurotransmitter function. Moreover, they suggest that exercise may increase cardiovascular health through changing central neural regulation of blood pressure. In spontaneously hypertensive rats (SHR), a deficiency in the inhibitory GABA neurotransmitter system within the posterior hypothalamic area contributes to tonically elevated levels of arterial blood pressure. Researchers identified a reduction in the GABA synthesizing enzyme glutamic acid decarboxylase (GAD) within the posterior hypothalamus of SHR [149]. Thus, exercise in the aged may further support the aging GABAergic system.

#### 2.25.8. Yoga, Music, and *γ*-Aminobutyric Acid

Yoga breathing has been shown to significantly increase GABA levels [150]. At least one study has also shown that certain relaxing music therapy acts via the GABA receptor and that this effect was blocked by the GABA receptor antagonists [151].

## 3. Conclusion

In conclusion, aging is associated with a decline in hormones and an associated decline in GABAergic inhibitory action or stimulatory action. This loss leads, in the case of GABA B receptors, to concomitant voltage-gated ion channel and calcium channel dysfunction. Neurosteroid hormones act as direct calcium channel blockers, or they act indirectly on calcium channels through their action on GABA receptors. The calcium channel dysfunction associated with hormone loss further leads to an excitatory cell state, which can ultimately lead to cell death. A decline in GABAergic function can be partially tied to decreases in progesterone, allopregnanolone, and DHEA associated with aging, vitamin deficiencies, and toxic chemicals in the food supply or environment. Hormones have binding sites on GABA receptors where they assist primarily with GABA receptor inhibitory functions. Thus, hormones, acting on GABA receptors, ultimately influence calcium channel regulation. The decline in GABAergic function associated with aging and a toxic environment has an effect on just about every body organ system, showing the universality of the importance of GABAergic function throughout the human body. Nutritional support of the GABAergic system may address some of the GABAergic dysfunction associated with aging and, thus, is a furtive area for further research.

## Data Availability

Data are available in the referenced reviewed publications.
